# The Molecular Cloning and Functional Analysis of the *FAD2* Gene in *Hippophe rhamnoids* L.

**DOI:** 10.3390/plants13223252

**Published:** 2024-11-20

**Authors:** Di Cong, Chang Ni, Luwen Han, Jianlin Cheng, Wei An, Siyu An, Hongzhang Liu, Huijing Liu, Dan Yao, Yuqin Fu, Shuying Liu, Guoshuang Chen

**Affiliations:** 1 College of Life Sciences, Jilin Agricultural University, Changchun 130118, China; 18946771390@163.com (D.C.); nichang99@163.com (C.N.); 13293598171@163.com (L.H.); 18370423403@163.com (J.C.); liuhongzhang@jlau.edu.cn (H.L.); huijingliu@jlau.edu.cn (H.L.); dyao@jlau.edu.cn (D.Y.); yuqinfu@jlau.edu.cn (Y.F.); 2 Jilin Province Product Quality Supervision and Inspection Institute, Changchun 130103, China; 13677667717@163.com (W.A.); ansiyu1991@163.com (S.A.); 3 Northeast Institute of Geography and Agroecology, Chinese Academy of Sciences, Changchun 130102, China

**Keywords:** fatty acid, *Hippophae rhamnoides* Linn., fatty acid desaturase, acetyl-acetyl carrier protein thioesterase

## Abstract

Seabuckthorn (*Hippophae rhamnoides* Linn.) is a commonly utilized medicinal crop with various applications in the treatment of different diseases. Two particularly noteworthy nutrients in seabuckthorn fruit are seabuckthorn oil and flavonoids. In recent years, seabuckthorn oil has attracted considerable attention due to its perceived benefits for beauty and healthcare. Consequently, there is a clear need for further research into seabuckthorn oil. While numerous studies have been conducted on the regulation of oil by the *FAD2* gene family, there is a paucity of literature examining the molecular mechanism of *FAD2* gene involvement in seabuckthorn oil regulation. Accordingly, two *FAD2* genes have been identified in seabuckthorn, which are classified differently and perform distinct functions. Both genes are located in the endoplasmic reticulum. Following transient expression in seabuckthorn fruits, it was observed that *HrFAD2-1* and *HrFAD2-3* were capable of influencing the synthesis of α-linolenic acid, with *HrFAD2-1* additionally demonstrated to facilitate the synthesis of lysophosphatidic acid. All of the aforementioned genes have been observed to promote jasmonic acid (JA) synthesis. The heterologous transformation of *Linum usitatissimum* demonstrates that both *HrFAD2-1* and *HrFAD2-3* are capable of promoting plant growth. The *HrFAD2-1* gene was observed to significantly increase the content of major fatty acids in *Linum usitatissimum* Linn seeds, whereas the *HrFAD2-3* gene appeared to be primarily involved in the regulation of plant growth and development. In conclusion, a preliminary investigation into the functions of the *HrFAD2-1* and *HrFAD2-3* genes in fatty acid synthesis was conducted. This revealed that *HrFAD2-1* is closely associated with oleic acid synthesis and acts as a negative regulator. Furthermore, our findings will provide a foundation for subsequent investigations into the fatty acid synthesis pathway in *Hippophae rhamnoides* oil, offering a theoretical basis for subsequent studies at the molecular level.

## 1. Introduction

Seabuckthorn (*Hippophae rhamnoides* Linn.) is a homologous plant that is both a food and a medicinal source. Research has shown that it contains over 190 bioactive compounds [1]. Seabuckthorn is a nutritionally dense fruit, and its seeds, leaves, and fruits are particularly rich in protein. The berries of the seabuckthorn plant have a long history of use as food and medicinal ingredients in eastern countries. Of particular interest to researchers are its oil and its flavonoids, which are two of the important nutrients found in the fruit [2]. The fruit of the seabuckthorn plant is capable of accumulating two distinct types of oil, one derived from the pulp and the other from the seeds. The seeds, in particular, are rich in bioactive substances and have been identified as a potential functional substance [3]. The content of unsaturated fatty acids in seabuckthorn oil is relatively high, representing 68.15% of the total fatty acid composition in seabuckthorn fruit oil. In comparison with seabuckthorn fruit oil, seabuckthorn seed oil contains a higher concentration of unsaturated fatty acids, which has a significant impact on the quality and efficacy of seabuckthorn oil [4]. Polyunsaturated fatty acids are essential for animals [5], and thus the study of these acids in seabuckthorn oil may facilitate an understanding of, and potential improvements to, the composition of seabuckthorn oil, thereby enhancing its quality. Despite the existing body of research on seabuckthorn oil, there is a dearth of studies that employ molecular technology to examine this substance.

Fatty acids (FAs) represent a class of organic compounds that are among the most significant components of living organisms. They are involved in several essential biological processes, including the specificity and tissue immunity of organisms, as well as cell recognition [6]. Given their role as integral components of cell membranes, these molecules can be harnessed as energy sources, thereby providing the cells with the necessary energy to function. Alternatively, they can be transformed into signal molecules, which enable the transfer of information within the cell [7]. Among these, unsaturated fatty acids (UFAs) are of particular significance. It has been demonstrated that the plasma and blood concentrations of polyunsaturated fatty acids in children and adolescents with attention deficit hyperactivity disorder (ADHD) are significantly reduced. This evidence lends support to the proposition that unsaturated fatty acids present in plant-based foods are of paramount importance for human health and thus warrant further investigation [8].

Fatty acid desaturase (*FAD*) functions as a catalyst for the formation of double bonds at specific positions within the fatty acid chain, thereby producing unsaturated fatty acids. *FAD*s are of various types and perform different functions, yet they all play pivotal roles in the synthesis of individual unsaturated fatty acids. The *FAD2* genes, which encode the ∆12 fatty acid desaturase, are widely present in plants. Linoleic acid is synthesized by the insertion of cis double bonds into the C12 and C13 positions of oleic acid. The *FAD2* gene plays a crucial role in the synthesis of linoleic acid from oleic acid, thereby influencing the fatty acid composition of plants. The impact of these fatty acids on plant stress resistance growth and development is significant [9,10]. In 1994, Okuley et al. initially cloned an *FAD2* gene from *Arabidopsis thaliana*, which was subsequently expressed in roots, leaves, and seeds. This demonstrated that the gene is involved in the synthesis of oil [11]. In recent years, a substantial body of research has demonstrated that the function of the *FAD2* gene is primarily concerned with the influence of fatty acid composition in plants. Following the promotion or inhibition of *FAD2* gene expression in plants via various biotechnological methods, alterations in the fatty acid composition ratio can be observed, these result from changes in fatty acids such as oleic acid, linoleic acid, and palmitoleic acid. The use of CRISPR/Cas9 technology has enabled the generation of a non-transgenic double mutant (*FAD2-1a*/*FAD2-1b*) with a high oleic acid content and which exhibits a significantly lower oleic acid content than that observed in the wild type [12]. A comparative analysis of the fatty acid content of recombinant *Saccharomyces cerevisiae* overexpressing the *OLE1* and *FAD2* genes revealed a higher concentration of fatty acids in these samples than in wild-type *Saccharomyces cerevisiae* pY15TEF1. Similarly, overexpression of the *SoFAD2* gene in yeast was found to result in an increased linoleic acid (LA) content [13].

The current focus of research into seabuckthorn oil is on the investigation of its function and the optimization of extraction methods. In recent years, there has been growing interest in molecular-level research, making it crucial when seeking to investigate the molecular mechanism of fatty acids in seabuckthorn oil. While there are numerous studies on the regulation of oil by the *FAD2* gene family, there are only a limited number of reports on the molecular mechanism of the *FAD2* gene involved in the regulation of seabuckthorn oil. In this study, we cloned two *HrFAD2* genes for the first time and conducted preliminary investigations into their expression patterns in *Hippophae rhamnoides*. Additionally, it was observed that both *HrFAD2-1* and *HrFAD2-3* have the capacity to stimulate plant growth through the heterologous transformation of mustard. The *HrFAD2-1* gene has been demonstrated to markedly enhance the concentration of principal fatty acids in flax mustard seeds. Additionally, the *HrFAD2-3* gene appears to be predominantly implicated in the regulation of plant growth and development. In conclusion, our results constitute a preliminary study on the function of two *HrFAD2* genes in regulating fatty acids, thereby providing a theoretical basis for subsequent studies of seabuckthorn oil at the molecular level and laying the foundation for the subsequent elucidation of the fatty acid synthesis pathway of seabuckthorn oil.

## 2. Results

### 2.1. Sequence Acquisition and Identification of the HrFAD2 Gene

**Figure 1:**
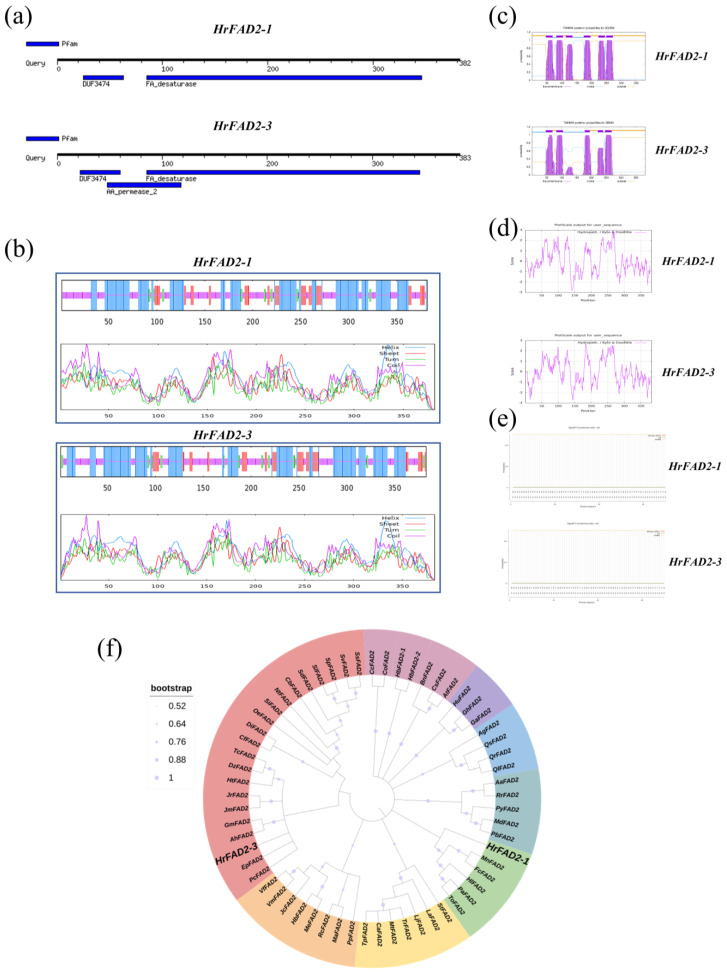
A bioinformatics analysis of the *HrFAD2* gene in *H. rhamnoides*. (**a**) A structural domain prediction was conducted on *HrFAD2-1* and *HrFAD2-3*. The predicted results were obtained from the website https://www.genome.jp/tools/motif/ (accessed on 15 September 2024). The images depict *HrFAD2-1* and *HrFAD2-3*, respectively, from **top** to **bottom**. (**b**) Secondary structure prediction of *HrFAD2-1* and *HrFAD2-3* proteins. The predicted results were obtained from the website https://npsa-prabi.ibcp.fr/ (accessed on 15 September 2024). The images show *HrFAD2-1* and *HrFAD2-3*, respectively, from **top** to **bottom**. (**c**) The transmembrane regions of the *HrFAD2-1* and *HrFAD2-3* proteins were predicted. The predicted results were obtained from the following website: http://www.cbs.dtu.dk/services/TMHMM/. The images show *HrFAD2-1* and *HrFAD2-3*, respectively, from **top** to **bottom**. (**d**) A prediction of the hydrophilicity of the *HrFAD2-1* and *HrFAD2-3* proteins was also made. The predicted results were obtained from the following website: https://web.expasy.org/protscale/ (accessed on 15 September 2024). The images show *HrFAD2-1* and *HrFAD2-3*, respectively, from **top** to **bottom**. (**e**) The following section presents the results of the signal peptide prediction for the *HrFAD2-1* and *HrFAD2-3* proteins. The predicted results were obtained from the website http://www.cbs.dtu.dk/services/SignalP/ (accessed on 15 September 2024). The images show *HrFAD2-1* and *HrFAD2-3*, respectively, from **top** to **bottom**. (**f**) An evolutionary tree analysis of *HrFAD2-1* and *HrFAD2-3*. The predicted results were obtained from the following website: https://www.ncbi.nlm.nih.gov/ (accessed on 15 September 2024).

All sequences of the *HrFAD2* gene family were retrieved from the RNA-seq database of seabuckthorn in the pre-laboratory stage. A total of 16 sequences were identified, and incomplete sequences in the coding sequence (CDS) region were removed based on the National Centre for Biotechnology Information Database. The *FAD2* gene sequences from other species were compared using the NCBI database (www.ncbi.nlm.nih.gov (accessed on 15 September 2024)). Two *HrFAD2* gene sequences with complete structural domains, high homology, and similar lengths were selected for further analysis. These were named *HrFAD2-1* and *HrFAD2-3*. The *HrFAD2-1* and *HrFAD2-3* genes were cloned from seabuckthorn fruits and encoded to the 382 and 383 amino acids, respectively. The structural domains of the target genes were analyzed using an online website (https://www.genome.jp/tools/motif/ (accessed on 15 September 2024)) to ascertain their classification. This revealed that *HrFAD2-1* and *HrFAD2-3* both belong to the *FAD2* gene family but differ in that *HrFAD2-3* has an additional structural domain of the permease, the carrier protein (Figure 1a).

The protein secondary structures of the target genes were predicted using an online website (http://npsa-prabi.ibcp.fr (accessed on 15 September 2024)). The results were found to demonstrate that the protein encoded by *HrFAD2-1* exhibited a composition of 42.41% α-helix, 41.88% irregular coil, 11.78% extended strand, and 3.93% β-turn. The protein encoded by *HrFAD2-3* has been found to have 44.13% α-helix, 37.60% irregular coil, 12.79% extended strand and 5.48% β-turn (Figure 1b). The transmembrane regions of both genes were investigated using online software (http://www.cbs.dtu.dk/services/TMHMM/, https://web.expasy.org/protscale/, https://services.healthtech.dtu.dk/services/SignalP-5.0/ (accessed on 15 September 2024)), which enabled the prediction of hydrophilicity and signal peptides. The results were found to demonstrate that *HrFAD2-1* possesses six transmembrane structural domains, while *HrFAD2-3* exhibits five transmembrane structural domains (Figure 1c). The hydrophilicity analysis demonstrated that both genes are hydrophilic proteins (Figure 1d) and that neither exhibited a signal peptide (Figure 1e). The homologous amino acid sequences of *HrFAD2* proteins in oilseed crops, including soybean, date palm, castor bean, rapeseed, coconut, sesame, mulberry and *Olea europaea*, as well as in *Arabidopsis thaliana* and *Linum usitatissimum* model plants, were obtained from the National Centre for Biotechnology Information Database (NCBI: https://www.ncbi.nlm.nih.gov/ (accessed on 15 September 2024)). A phylogenetic tree was constructed using MEGA-X software. The resulting phylogenetic tree indicated that *HrFAD2-1* was the closest homologue to the coconut *FAD2* gene, while *HrFAD2-3* was the closest relative to the mulberry *FAD2* gene (Figure 1f).

### 2.2. Subcellular Localization Analysis

**Figure 2:**
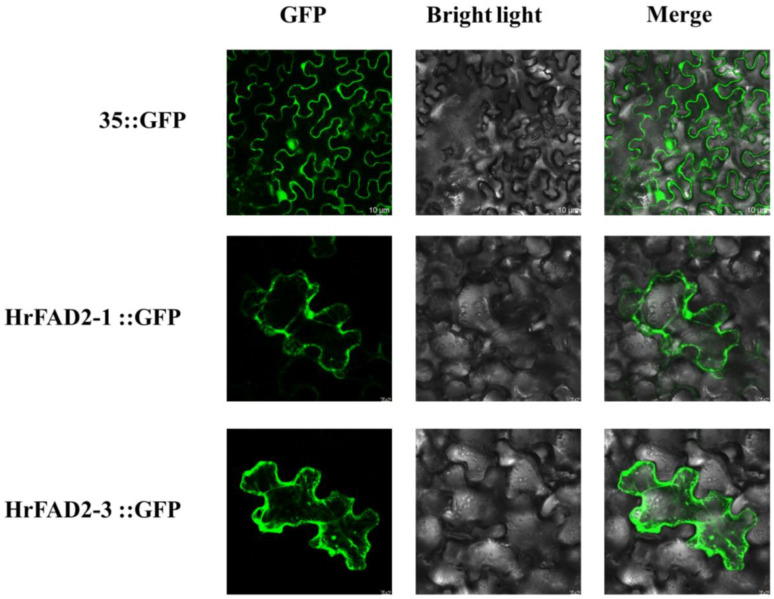
Results of the subcellular localization of *HrFAD2-1* and *HrFAD2-3*. Blank control 35s–GFP null and HrFAD2-1–GFP and HrFAD2-3–GFP fusion proteins were transiently expressed in tobacco leaves and visualized by confocal microscopy after 72 h. The images displayed on the **left**, **middle**, and **right** represent those captured in the dark, bright, and merge attempts, respectively.

Subcellular localization can localize a protein or expression product to specific locations in the cell, such as the nucleus, various organelles and the plasma membrane, and thus provide research avenues for understanding the mechanism of action of genes. To determine the subcellular localization of *HrFAD2-1* and *HrFAD2-3* proteins, we constructed recombinant vectors containing green fluorescent protein (GFP) and injected them into 3–4-week-old tobacco leaves. The samples were then treated in the dark for 72 h and GFP fluorescence was detected using a scanning confocal microscope. The results show that both *HrFAD2-1-* and *HrFAD2-3*-expressed proteins showed fluorescent signals in the endoplasmic reticulum (Figure 2).

### 2.3. Analysis of the Expression Pattern of the HrFAD2 Gene

**Figure 3:**
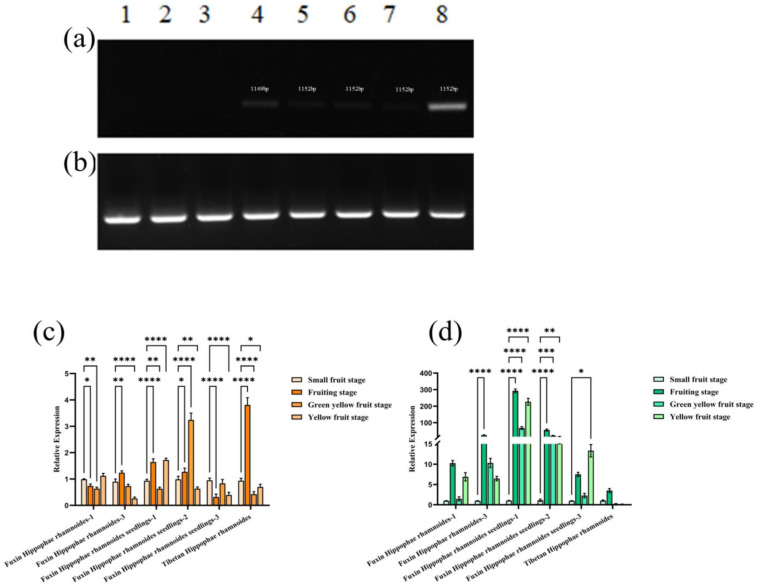
Analysis of the expression pattern of the *HrFAD2* gene in different trees. Semi-quantitative preliminary determination of the expression pattern of *HrFAD2-1* as well as *HrFAD2-3* genes. (**a**) Expression in different tissue sites: 1–4 are *HrFAD2-1* genes, 5–8 are *HrFAD2-3* genes, and from left to right are leaves, roots, stems, and fruits, respectively. (**b**) Endogenous gene *HrGAPDH*. (**c**) Analysis of the expression pattern of *HrFAD2-1* gene in different trees and at different periods of fruit development. (**d**) Analysis of the expression pattern of the *HrFAD2-3* gene in different trees and at different periods of fruit development (*: *p* < 0.05, **: 0.001 < *p* < 0.01, ***: 0.0001 < *p* < 0.001, ****: 0.00001 < *p* < 0.0001).

To gain insight into the pivotal function of the *HrFAD2* gene in seabuckthorn fruits, we conducted an analysis of the expression levels of the *HrFAD2-1* and *HrFAD2-3* genes in the leaves, roots, stems, and fruits of seabuckthorn trees. Initially, it was observed that both the *HrFAD2-1* and *HrFAD2-3* genes were expressed in the fruits, with the *HrFAD2-3* gene also exhibiting expression in the leaves, stems, and roots (Figure 3a,b). It was postulated that the *HrFAD2-3* gene may also exert a modulating influence on plant growth and development.

The fruit of *Hippophae rhamnoides* can be divided into four distinct stages, namely the small fruit stage, green fruit stage, yellow fruit stage, and ripe fruit stage, in accordance with the varying growth stages. In order to investigate the expression levels of the *HrFAD2-1* and *HrFAD2-3* genes in different varieties and at different periods, two Fuxin *Hippophae rhamnoides*, three Fuxin *Hippophae rhamnoides* seedlings and one Tibetan *Hippophae rhamnoides* were selected as samples for analysis. The results demonstrate that the expression level of *HrFAD2-1* in Fuxin *Hippophae rhamnoides* exhibited variability across different varieties, with distinct trends observed between strains within the same variety. The expression trend of Fuxin *Hippophae rhamnoides* no. 3 exhibited a close correlation with that of Tibetan *Hippophae rhamnoides* no. 1, with the highest expression level observed at the green fruit stage, followed by a decline in expression with fruit ripening. In both Fuxin *Hippophae rhamnoides* and Fuxin *Hippophae rhamnoides* seedlings, the expression of *HrFAD2-1* was highest at the yellow fruit stage. However, there was a notable discrepancy in the subsequent decline in expression, with *HrFAD2-1* in Fuxin *Hippophae rhamnoides* gradually decreasing from the small fruit stage to the green fruit stage, while, in Fuxin *Hippophae rhamnoides*, expression increased at the green fruit stage. It is notable that the trend observed in the Fuxin *Hippophae rhamnoides* seedling progeny numbers 2 and 3 differs from that of other trees. The highest expression levels were observed for no. 2 in the green and yellow fruit stages, while no. 3 exhibited the highest expression levels in the small fruit stage. However, both exhibited a significant increase in the green and yellow fruit stages (Figure 3c).

Nevertheless, the expression pattern of the *HrFAD2-3* gene differs from that of the *HrFAD2-1* gene. The expression level of *HrFAD2-3* is highly consistent across different varieties and lines of the same variety, with the highest expression occurring at the green fruit stage. As the fruit matures, the expression level of the gene also declines. Among the varieties, Fuxin *Hippophae rhamnoides* no. 1 and Fuxin *Hippophae rhamnoides* seedling no. 1 exhibited a slight increase during the yellow fruit period, while Fuxin *Hippophae rhamnoides* seedling no. 3 demonstrated the highest expression level during this phase. As with other cultivars, Fuxin *Hippophae rhamnoides* seedling no. 3 exhibited a high expression level during both the green fruit period and the yellow fruit period. However, the expression level decreased significantly during the small fruit period and the green fruit period (Figure 3d).

### 2.4. Transient Transformation of HrFAD2 Gene in Hippophae rhamnoides Fruit

**Figure 4:**
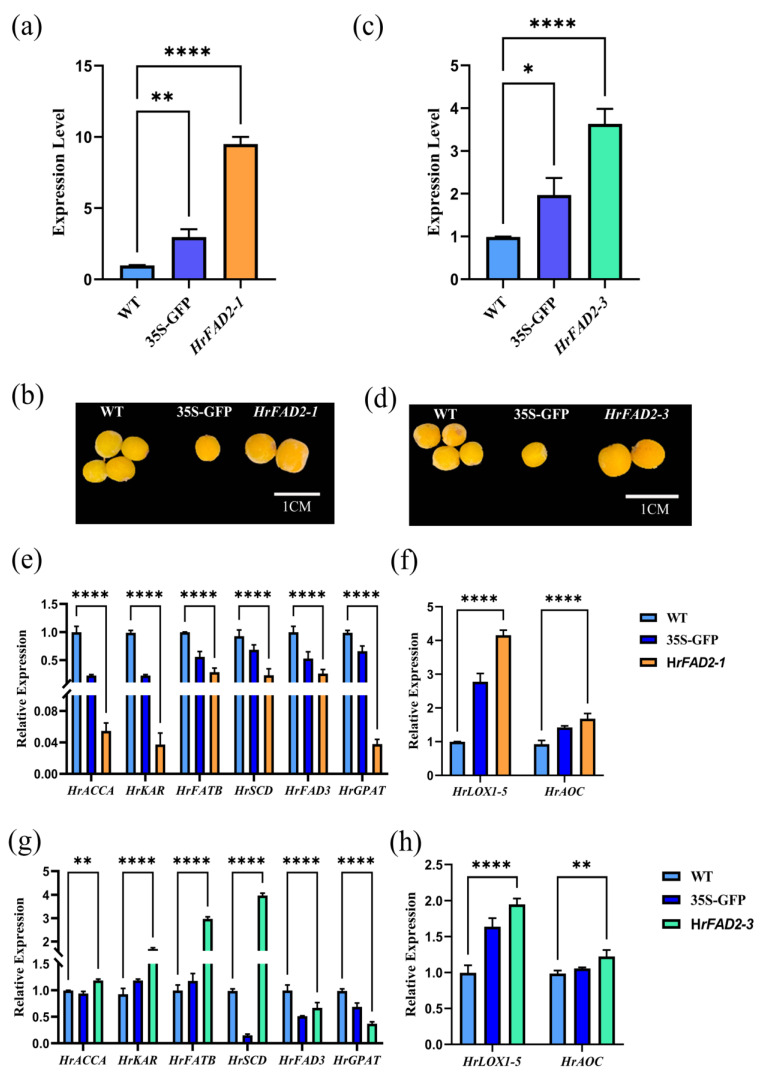
Phenotypic observation and expression analysis of *HrFAD2-1* as well as *HrFAD2-3* genes transiently transformed in seabuckthorn fruits. (**a**,**c**) Elevated expression of *HrFAD2-1* and *HrFAD2-3* genes after transient transformation in seabuckthorn fruits. (**b**,**d**) Phenotypic observations of *HrFAD2-1* as well as *HrFAD2-3* genes after transient transformation in seabuckthorn fruits. (**e**) Changes in gene expression in the fatty acid synthesis pathway in *HrFAD2-1*-gene-transformed fruit. (**f**) Changes in gene expression in the JA pathway in *HrFAD2-1*-gene-transformed fruit. (**g**) Changes in gene expression in the fatty acid synthesis pathway in *HrFAD2-3*-gene-transformed fruit. (**h**) Changes in gene expression in the JA pathway in *HrFAD2-3*-gene-transformed fruit (WT: seabuckthorn without any treatment, 35s-GFP: seabuckthorn transferred to empty loads, *: *p* < 0.05, **: 0.001 < *p* < 0.01, ****: 0.00001 < *p* < 0.0001).

The term ‘transient expression’ is used to describe the rapid accumulation of total protein in plants following the transfer of exogenous genes. This is achieved through the processes of transcription and translation, which can be employed for the expeditious analysis of target genes. To further elucidate the functions of the *HrFAD2-1* and *HrFAD2-3* genes in *Hippophae rhamnoides*, a transient transformation of the fruits of this species was conducted. According to the findings of previous laboratory research, the accumulation of fatty acids in seabuckthorn fruit commences during the green fruit stage and reaches its peak after the yellow fruit stage. Consequently, the decision was taken to inject at the green fruit stage. The results of the quantitative real-time PCR (qRT-PCR) were found to demonstrate that the expression levels of the wild-type fruits, pCAMBIA1300 empty fruits, pCAMBIA1300-HrFAD2-1, and pCAMBIA1300-HrFAD2-3 exhibited a gradual increase. In comparison with the wild type, the expression levels in the OE group exhibited a notable alteration, thereby substantiating the efficacy of the treatment (Figure 4a,c).

The results demonstrate that the fruits treated with pCAMBIA1300-HrFAD2-1 exhibited a significantly larger size than the wild-type and pCAMBIA1300 empty-load fruits. Additionally, the fruits treated with pCAMBIA1300-HrFAD2-1 displayed a notable yellower hue and a larger size when compared with the wild-type fruits (Figure 4b). In comparison with the wild-type fruits, the pCAMBIA1300-HrFAD2-3 fruits exhibited a less pronounced change in size; however, they displayed a notable yellowish hue, differing from that of the wild-type fruits (Figure 4d).

By analyzing the expression levels of related enzyme genes, it was found that the expression levels of *HrACCA*, *HrKAR*, *HrFATB*, and *HrSCD* were significantly lower than those of the wild type in the group treated with pCAMBIA1300-HrFAD2-1 (Figure 4e). Furthermore, the expression levels of *HrACCA*, *HrKAR*, *HrFATB*, and *HrSCD* were significantly higher than those of the wild type (Figure 4g). The expression levels of *HrFAD3* and *HrGPAT* were found to be significantly lower than those of the wild type in the pCAMBIA1300-HrFAD2-1 treatment group and the pCAMBIA1300-HrFAD2-3 treatment group among the key enzyme genes downstream of oleic acid synthesis. It was therefore hypothesized that the *HrFAD2-1* gene might affect the synthesis of γ-linolenic acid in seabuckthorn fruits, whereas the *HrFAD2-3* gene had an inhibitory effect on the ab initio synthesis of plant fatty acids.

The expression of *HrLOX1-5* and *HrAOC* was markedly elevated in the treated group relative to the wild type, suggesting that the *HrFAD2-1* and *HrFAD2-3* genes exert a stimulatory influence on jasmonic acid (JA) synthesis (Figure 4f,h). It can be postulated that *HrFAD2-1* and *HrFAD2-3* are involved in jasmonic acid (JA) synthesis in the plant species *Hippophae rhamnoides* and that they play a role in the cold tolerance of this species.

### 2.5. Overexpression of HrFAD2 Gene in Flax Mustard

To further study the function of the *HrFAD2* gene, we constructed overexpression recombinant vectors pCAMBIA3301-HrFAD2-1 and pCAMBIA3301-HrFAD2-3. Agrobacterium-mediated transformation was used to introduce them into mustard and overexpressed strains were obtained. After preliminary screening with Basta, the presence of transgenic lines was further verified by PCR. Next, high-expression lines were screened by qRT-PCR and at least nine transgenic lines of pCAMBIA3301-HrFAD2-1 and pCAMBIA3301-HrFAD2-3 were ultimately obtained (Supplementary Figure S1a,b). Phenotypic observation showed that the transgenic plants of pCAMBIA3301-HrFAD2-1 and pCAMBIA3301-HrFAD2-3 were stronger and had larger leaves than wild flax (Supplementary Figure S1c,d).

**Figure 5:**
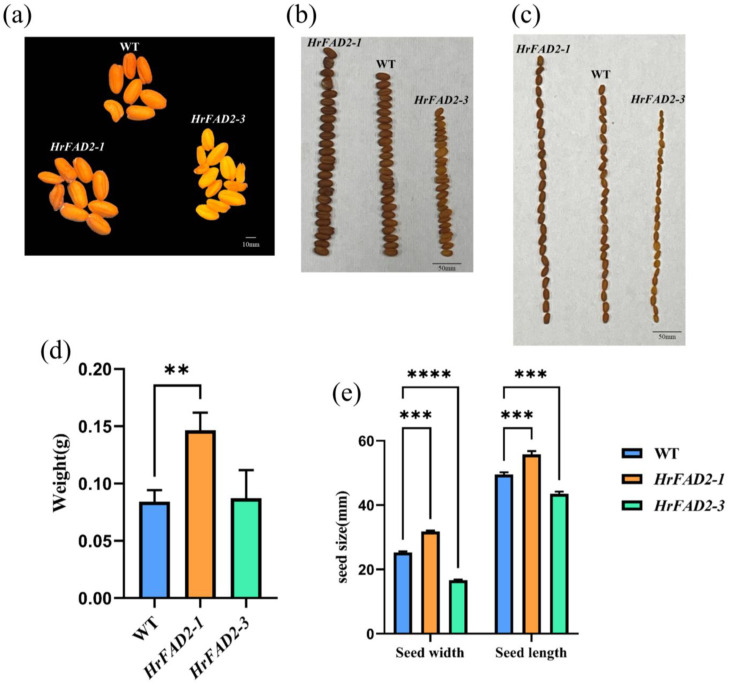
Observations on the seed phenotype of *HrFAD2*-gene-positive plants. (**a**) Comparison of seed color. (**b**) Comparison of seed width. (**c**) Comparison of seed length. (**d**) Comparison of 100-seed weight. (**e**) Comparison of statistical data on seed length and width (WT: flax mustard without any treatment, **: 0.001 < *p* < 0.01, ***: 0.0001 < *p* < 0.001, ****: 0.00001 < *p* < 0.0001).

The phenotype of the seeds was observed using a stereomicroscope. Compared with wild-type linseed, pCAMBIA3301-HrFAD2-1 transgenic linseed was larger and darker in color, whereas pCAMBIA3301-HrFAD2-3 transgenic linseed was smaller and darker in color (Figure 5a). The length and width of wild-type linseed are larger than those of pCAMBIA3301-HrFAD2-3 transgenic seeds and smaller than those of pCAMBIA3301-HrFAD2-1 transgenic seeds (Figure 5b,c,e).

Three groups of transgenic linseed and three groups of wild linseed were randomly selected to compare the 100-seed weight. The results were found to show that, compared with the 100-seed weight of wild linseed, the 100-seed weight of pCAMBIA3301-HrFAD2-1 transgenic seeds increased significantly (Figure 5d), while the 100-seed weight of pCAMBIA3301-HrFAD2-3 transgenic seeds did not change significantly (Figure 5d).

### 2.6. The HrFAD2 Gene Is Involved in Oil Synthesis

**Figure 6:**
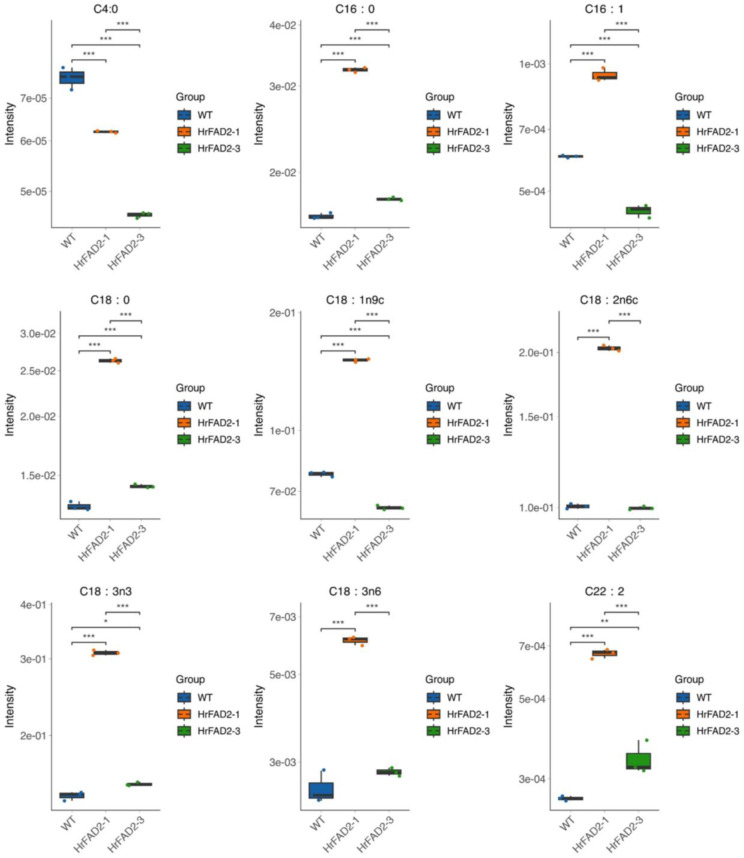
Detection of fatty acid content in *HrFAD2* transgenic flaxseed mustard. C4:0: Methyl butyrate. C16:0: Methyl palmitate. C16:1: Methyl palmitoleate. C18:0: Methyl stearate. C18:1n9c: Methyl oleate. C18:2n6c: Methyl linoleate. C18:3n3: Methyl linolenate. C18:3n6: Methyl r-linolenate. C22:2: cis-13,16-docosadienoic acid (*: *p* < 0.05; **: 0.001 < *p* < 0.01; ***: 0.0001 < *p* < 0.001).

We also investigated the role of the *HrFAD2* gene in lipid synthesis, as it strongly influences the quality and efficacy of linseed oil. GC–MS was used to analyze the major fatty acid content of transgenic linseed. The recoveries of fatty acid content ranged from 86%~97% with high accuracy. In addition, all standards’ relative standard deviations (RSDs) were less than 30%, indicating good reproducibility. These results demonstrate the good reliability of the method we used. Next, we found that overexpression of the *HrFAD2-1* and *HrFAD2-3* genes could alter the fatty acid ratio in linseed. Overexpression of the *HrFAD2-1* gene significantly increased the levels of the major fatty acids in linseed, with the exception of methyl butyrate. In contrast, overexpression of the *HrFAD2-3* gene had little effect on the fatty acid content of linseed (Figure 6).

## 3. Discussion

*Hippophae rhamnoides* is widely used as a medicinal and food plant. *Hippophae rhamnoides* oil is rich in bioactive compounds and has been recognized as a potential functional substance. In this study, we cloned, for the first time, two *FAD2* genes in *Hippophae rhamnoides*, namely *HrFAD2-1* and *HrFAD2-3*. The bioinformatics analysis of *HrFAD2-1* and *HrFAD2-3* showed that they were highly consistent in terms of the physical and chemical properties of the protein, subcellular localization prediction, secondary structure of the protein, hydrophilicity analysis, and signal peptide prediction, which also proved the conservatism of the genes. The amino acid sequence length of soybean *GmFAD2-2* and *GmFAD2-3* is 383 [14] and the subcellular localization of the *FAD2* gene in oilseed rape and sunflower is in the endoplasmic reticulum. At the same time, in the secondary structure of the protein, α-helix and random curl account for a large proportion [15]. Additionally, maize *FAD2* is a hydrophilic protein without a signal peptide [16], which is consistent with the experimental results in this paper. In addition to the fatty acid desaturase domain, *HrFAD2-3* has another osmotic enzyme domain, the carrier protein, when compared with *HrFAD2-1*. At the same time, the phylogenetic tree constructed shows that *HrFAD2-1* has the closest homology with the coconut *FAD2* gene, while *HrFAD2-3* has the closest genetic relationship with the mulberry *FAD2* gene. The difference in genetic relationship and domain with other species proves that the functions of the two genes in *Hippophae rhamnoides* are not completely consistent. According to the classification of *FAD2* [17], it can be concluded that *HrFAD2-1* belongs to the constitutive expression and *HrFAD2-3* belongs to the seed-specific expression, which have different functions in *Hippophae rhamnoides*. *FAD2* can differ in many properties, such as the number of transmembrane domains and the length of conserved motifs in plants, among which olive and cotton have four and six transmembrane domains, respectively [18]. This is proven by the observation that *HrFAD2-1* has six transmembrane domains and *HrFAD2-3* has five transmembrane domains.

Unsaturated fatty acids in plants are essential for human health, and edible oils rich in unsaturated fatty acids are becoming increasingly popular, so studying unsaturated fatty acids in plants has a bright future. Plastids (or chloroplasts, as in immature seeds) are the main sites of fatty acid synthesis in plants [19]. Acetyl-CoA in the mitochondria is exported to the cytoplasm via the citrate–pyruvate cycle, where the final fatty acids are synthesized in the endoplasmic reticulum [20]. The end product of the saturated fatty acid synthesis pathway is soft fat [21]. Both *HrFAD2* were found to be located in the endoplasmic reticulum, as shown by the subcellular localization results in this experiment.

Even in the same plant, the *FAD2* gene has different functions with different expression patterns [22]. According to the specificity of tissue expression, the *FAD2* gene can be divided into seed-specific expression and constitutive expression [23]. For example, in soybean, *FAD2-1A* and *FAD2-1B* are seed-specific expressions that mainly regulate oleic acid to produce linoleic acid; *FAD2-2* and *FAD2-3* are mainly responsible for the synthesis of unsaturated fatty acids in membrane lipids in vegetative tissues and seeds and are constitutive expressions [24,25,26]. In this study, we used qRT-PCR to analyze the expression patterns of the three genes in *Hippophae rhamnoides* in order to facilitate the future study of the genes. Semi-quantitative results were found to show that *HrFAD2-1* was expressed only in fruits, while *HrFAD2-3* was expressed in leaves, stems, roots and fruits, and that the highest expression level was found in fruits, which is consistent with the expression pattern of the *Rhus verniciflua FAD2* gene, indicating that the expression sites of *HrFAD2-1* and *HrFAD2-3* genes in *Hippophae rhamnoides* plants are different. In conclusion, according to the classification of the *FAD2* gene family, *HrFAD2-1* may belong to the seed-specific expression and play an important role in fatty acid synthesis, while *HrFAD2-3* belongs to the constitutive expression, which plays a role not only in fatty acid synthesis but also in plant growth and development.

The oil content of Fuxin *Hippophae rhamnoides* increased rapidly at the green fruit stage and then stabilized with fruit ripening, whereas the oleic acid content did not change regularly with fruit ripening. Therefore, it is speculated that *HrFAD2-1* is closely related to oleic acid content and that fatty acid composition can be altered by controlling oleic acid content, whereas *HrFAD2-3* may be related to the total amount of fatty acids. We quantified the key enzyme genes of the fatty acid synthesis pathway and the gene treatment groups of *HrACCA*, *HrKAR*, *HrFATB*, and *HrSCD* were found to be significantly lower than the wild type. After Zhou et al. transformed *HaFAD2-1* by heterologous expression, the total unsaturated fatty acid content of *Arabidopsis* seeds and most of the fatty acids downstream of linoleic acid synthesis increased significantly, while most of the fatty acids upstream of linoleic acid synthesis decreased significantly, indicating that *HaFAD2-1* may be an important gene involved in unsaturated fatty acid synthesis in sunflower [27]. This shows that *HrFAD2-1* is closely related to oleic acid synthesis and has a negative regulatory relationship. Δ6 fatty acid desaturase (FAD3) is the key enzyme in the synthesis of alpha-linolenic acid [28]. The expression level of the *HrFAD3* gene in the treatment group is lower than that of the wild type, indicating that the *HrFAD2-1* gene may affect the synthesis of γ-linolenic acid in the fatty acid desaturase pathway of *Hippophae rhamnoides*. *HrGPAT* is the key enzyme gene for the synthesis of lysophosphatidic acid from glyceryl triphosphate [29], suggesting that *HrFAD2-1* promotes lysophosphatidic acid synthesis. After treatment with pCAMBIA1300-HrFAD2-3, the key enzyme genes of the fatty acid synthesis pathway, namely *HrACCA*, *HrKAR*, *HrFATB*, and *HrSCD*, were found to be significantly higher in the treatment group than in the wild type, but the expression of *HrFAD3*, *HrGPAT* genes was lower than in the treatment group. Wu transformed *Arabidopsis thaliana* with the *PfFAD3.1* gene from perilla by soaking in flowers and eventually found *ACCA* and *FAT*. The key enzyme genes for fatty acid synthesis and accumulation, such as *FAD2* and *DGAT*, and the gene expression of *WRI1*, *ABI3*, and *FUS3*, which are involved in seed development, were found to be upregulated. *PfFAD3.1* may be involved in the regulation of seed growth and development by regulating the metabolic flux of fatty acids, which is similar to our experimental results [29]. It is speculated that *HrFAD2-3* may affect plant growth and development by participating in the synthesis of fatty acids.

In addition to altering the fatty acid composition of plants, the *FAD2* gene also has some influence on plant stress resistance, making plants more resistant to cold and salt. Overexpression of *OLE1* and *FAD2* genes has been found to result in recombinant *Saccharomyces cerevisiae* cells that are more resistant to multiple stresses and exhibit better membrane functions, including membrane fluidity and integrity [30]. Temperature has tissue specificity in regulating oleic acid desaturation at the transcriptional level. *CtFAD2-1*, *CtFAD2-2*, and *CtFAD6* significantly induced young leaves under cold and heat stress, whereas *CtFAD2-2* and *CtFAD6* slightly induced young stems [31]. In our research, through quantitative analysis, it was found that the expression levels of *HrLOX1-5* and *HrAOC* were significantly higher than those of the wild type, and that the important step of jasmonic acid synthesis catalyzed by *AOC* showed that *HrFAD2-1* and *HrFAD2-3* could promote the synthesis of JA [32]. Additionally, *LuFAD2A* and *LuFAD3A* in flax was found to promote the biosynthesis of jasmonic acid by increasing the content of linolenic acid. It can be concluded that *HrFAD2-1* and *HrFAD2-3* are involved in the JA synthesis of *Hippophae rhamnoides*, which has some effect on the cold tolerance of *Hippophae rhamnoides* [33]. Fan et al. analyzed the differential expression of the transcriptome in two stages of the seed development of high- and low-linoleic acid safflower varieties and identified 47 genes related to lipid biosynthesis. *FAD* genes such as *FAD2* (Chr8G0104100), *FAD3*, *FAD7*, and *FAD8* were found to promote the consumption of oleic acid conversion, and the coordinated regulation of these genes ensures the high accumulation of oleic acid in safflower seed oil [34]. Jiang et al. expressed five members of the *ZjFAD2* family in tobacco and their results show that all five genes increased linoleic acid levels [35]. Through transcriptional and metabolic analysis, He et al. found that two FAD2 homologous genes from *Cornus officinalis Siebold & Zucc.* may play a key role in controlling the ratio of oleic acid to linoleic acid [36]. Miao et al. showed that only three types of AsFAD2 (*Cephalosporium globosum*) enzymes (AsFAD2-1, -10, and -23) are δ12-oleate desaturases capable of converting oleic acid to linoleic acid, while *AsFAD2-1* and *AsFAD2-10* can also produce palmitoleic acid [37]. In our study, overexpression of the *HrFAD2-1* and *HrFAD2-3* genes can alter the fatty acid ratios in linseed. Overexpression of the *HrFAD2-1* gene can significantly increase the content of the major fatty acids in linseed, though methyl butyrate and other fatty acids are significantly increased. Acetyl-CoA is required for the synthesis of butyric acid, which competes with the de novo synthesis of fatty acids, thus reducing the level of butyric acid. Overexpression of the *HrFAD2-3* gene did not significantly change the fatty acid content of linseed, which is consistent with the experimental results of the transient transformation of *Hippophae rhamnoides* in the previous period, which show that *HrFAD2-3* has little effect on the fatty acid content of plants and may be involved in the regulation of plant growth and development. To date, there have been many studies on the role of the *FAD2* gene in plant oil synthesis, but the mechanism of seabuckthorn oil synthesis is still unclear. In this study, by investigating the correlation between the expression of *HrFAD2* and oil synthesis, it was found that *HrFAD2-1* plays a regulatory role as a negative regulator in the oil synthesis of *Hippophae rhamnoides*. In addition, the quantitative expression of *HrFAD2* can promote the synthesis of JA, which may improve the cold hardiness of *Hippophae rhamnoides*.

In conclusion, we found two *FAD2* genes with different classifications in *Hippophae rhamnoides* L., among which *HrFAD2-1* is a negative regulator in the oleic acid synthesis pathway, and we found that both *HrFAD2-1* and *HrFAD2-3* play important roles in plant growth and development through heterologous transformation in linseed. Additionally, we found that the *HrFAD2-1* gene can significantly increase the content of major fatty acids in linseed. It is speculated that participation in JA synthesis in *Hippophae rhamnoides* may have some effect on the cold tolerance of *Hippophae rhamnoides*.

## 4. Materials and Methods

### 4.1. Plant Materials and Growth Conditions

Plants used in the experiments were obtained from three live progeny of Fuxin *Hippophae rhamnoides* seedlings no. 1, no. 2 and no. 3 trees, three live progeny of Fuxin *Hippophae rhamnoides* no. 1 and no. 3 trees and from fruits of Tibetan *Hippophae rhamnoides* from the seabuckthorn plantation at Jilin Agricultural University, Changchun City, Jilin Province, China (coordinates 43.810433° N, 125.410385° E), with fruits collected centrally from the small fruiting stage, green fruiting stage, green and yellow fruiting stage, and yellow fruiting stage. These were frozen in liquid nitrogen immediately after collection and stored at −80 °C for subsequent experiments.

*Linum usitatissimum* and *Tobacco Benedictus* were grown in an artificial climate chamber under conditions including a 16 h light and 8 h dark cycle at 22–25 °C, 4500 lux supplemental light, and 50% relative humidity.

### 4.2. Cloning of HrFAD2 Gene Sequence

Two raw sequences of the *HrFAD2* gene were obtained according to the pre-laboratory seabuckthorn RNA-seq database (login number: PRJNA612989). The cDNA of seabuckthorn was used as the template for screening the optimal annealing temperature. Five minutes of pre-denaturation was performed at 94 °C, followed by denaturation at 94 °C for 30 s, annealing at 51–60 °C for 30 s, and extension at 72 °C for 1 min for 30 cycles. Ten minutes of extension at 72 °C was used as the last cycle of the procedure, and the optimal annealing temperature was determined by the depth of the target bands of the PCR products. Specific primers were designed using Primer 7.0 (Table S1).

The PCR products were purified by Agarose Gel Extraction Kit (TaKaRa Bio, Beijing, China), and the purified target gene products were ligated with the cloning vector pMD-18T (TaKaRa Bio, Beijing, China) and transformed into Escherichia coli DH5α (AngYuBio, Shanghai, China) in order to select a single colony for bacteriological PCR and sequencing (Sangon Biotech, Shanghai, China). The sequence comparison of target genes was performed by DNAMAN 8.0 software.

### 4.3. Bioinformatics Analysis

The secondary structure, hydrophilicity analysis, domain analysis, signal peptide analysis and transmembrane region prediction of *HrFAD2* gene are listed in Table S2 as Supplementary Data.

### 4.4. Phylogenetic Analysis

Other *FAD2* protein sequences are from NCBI and plant communities in https://www.ncbi.nlm.nih.gov/ (https://www.plantplus.cn/cn (accessed on15 September 2024)). The phylogenetic tree analysis of *HrFAD2* gene was carried out by MEGA-X software using the proximity method.

### 4.5. RNA Extraction and qRT-PCR Analysis

A column-type plant total RNA extraction and purification kit (Vazyme, Nanjing, China) was used. Next, the purified total RNA (0.5–2 μg) was reverse transcribed into first-strand cDNA for RT-PCR and qRT-PCR analyses using the Reverse Transcription Kit (TaKaRa Bio, Beijing, China). The qRT-PCR analysis was performed using Magic SYBR Mixture (Cwbio, Beijing, China) and all experiments were repeated more than three times. Gene-specific primers were designed using Primer 7.0 (Table S1).

### 4.6. Subcellular Localization Analysis of HrFAD2 Gene

The target genes were amplified using specific primers containing restriction sites (Table S1), and the obtained target genes were ligated into pCAMBIA1300 expression vectors containing the 35S promoter and EGFP progenitor using T4 ligase (TAKALA). The constructed pCAMBIA1300-FAD2-1 and pCAMBIA1300-FAD2-3 vectors were introduced into Agrobacterium EHA105 for transient expression. Agrobacterium containing the target gene was inoculated using YEP/Kanamycin/Acetosyringone (1000/1/1, *v*/*v*/*v*) liquid medium, and, after shaking the incubator at 28 °C overnight, the organisms were retained by centrifugation at 4 °C, 5000 rpm for 20 min. The bacteria were resuspended using a liquid mixture of 10 mM MgCl/10 mM MES (1/1, *v*/*v*), and the organisms were obtained by resting at room temperature for 2–3 h. The infiltration solution, with an OD value of 0.4–0.6, was used to transiently infiltrate 3–4-week-old *T. benthamiana* and incubated in the dark for 72 h. Observations were made using a laser confocal microscope.

### 4.7. Development of Transgenic Flax Mustard with HrFAD2

The target genes were amplified using specific primers containing restriction sites (Table S1) and the obtained target genes were ligated into pCAMBIA3301 expression vectors containing the 35S promoter using T4 ligase (TAKALA). Next, the constructed pCAMBIA3301-FAD2-1 and pCAMBIA3301-FAD2-3 vectors were introduced into Agrobacterium EHA105. The inflorescence infestation method was used to genetically transform *Linum usitatissimum,* and the phenotype and gene expression of the positive plants were subsequently tested to verify the function of the target genes.

### 4.8. Transient Expression of HrFAD2 Gene in Hippophae rhamnoides Fruit

According to previous laboratory studies, seabuckthorn fruits begin to accumulate fatty acids at the green fruit stage and the accumulation reaches a maximum after the yellow fruit stage. Therefore, for this experiment we chose to transiently transform at the green fruit stage and harvest at the yellow fruit stage. Configuration of the infestation solution was carried out according to the section “Subcellular Localization Analysis of *HrFAD2* Gene” above. The infestation solution was injected from the base of the fruit into the fruit using a sterile syringe (avoiding the seeds when injecting) and the fruits were collected for further testing after approximately 60-70 days. These experiments included analysis of target gene expression and expression of genes for fatty acid synthesis pathways and growth-and-development-related enzymes.

### 4.9. GC–MS Sample Preparation

Accurately weigh 0.5 g of freeze-dried seeds into a centrifuge tube, add 4 mL of chloroform solution, vortex and mix well; add 0.9% sodium chloride solution and vortex well. Isolate the core at 3500 rpm for 15 min, remove the substrate, transfer to a new centrifuge tube, add 2 mL of dichloromethane, mix well by vortexing, centrifuge for 15 min, remove the substrate, repeat the extraction twice, combine and mix well, dry with nitrogen, add 2 mL of methanol solution (including 5% sulfuric acid solution), mix well by vortexing. After cooling, add 2 mL of n-hexane solution, then add 1 mL of water solution, vortex mix for 30 s, add the supernatant to 1 mL of water solution, vortex mix for 30 s, centrifuge at 2000 rpm for 5 min, and add the supernatant to 1 mL of water solution. Vortex for 30 s, centrifuge at 2000 rpm for 5 min and collect the supernatant. The supernatant was blown dry in a nitrogen stream, an appropriate volume of isooctane solution was added, vortexed and on-line detection was performed after standing.

### 4.10. GC–MS Analysis

FA was analyzed by gas chromatography–mass spectrometry (GC–MS). The metabolites in the biological samples were analyzed qualitatively and quantitatively by comparison with information such as retention time, molecular mass (molecular mass error < 10 ppm), and then bioinformatic analysis. The Agilent GC system (Agilent 7820, Agilent Technologies, Santa Clara, CA, USA) is equipped with a CP-Sil 88 column (100 m × 0.25 mm × 0.25 µm, Agilent, Santa Clara, CA, USA). The GC conditions are as follows: the injection volume is 1 uL, the shunt ratio is 10:1, the carrier gas is high-purity helium at a flow rate of 1.0 mL/min, the initial diurnal night column chamber is 100 °C for 5.0 min, and the temperature is heated at 4 °C to 240 °C/min for 15 min. The mass spectrometer is an Agilent quadrupole mass spectrometry system (Agilent 5977, Agilent Technologies Inc., Santa Clara, CA, USA) equipped with an electron bombardment ion source (EI) and a MassHunter workstation. The inlet temperature is 260 °C and the quadrupole temperature is 150 °C. The scan mode is single-channel scanning (SIM) and the quality scan range is 30 to 550 *m*/*z*. Data were collected using MassHunter GC–MS Acquisition (Agilent Technologies, Inc., Santa Clara, CA, USA).

Qualitation of fatty acids was based on the accurate molecular mass of the compound determined by high-resolution mass spectrometry with a molecular mass error of <10 ppm, thus eliminating impurity interferences. Raw data were pre-processed using Quant-My-Way (Agilent Technologies) for baseline filtering, peak identification, peak matching, retention time correction, peak alignment, etc. to produce a data matrix containing retention times, mass-to-charge ratios, and peak intensities, which was used to confirm individual components.

Quantification of fatty acids was determined as follows: Quant-My-Way (Agilent Technologies) was used to fit the standard curve to obtain the absolute quantification of the compounds by the external standard method. The raw data exported by the software were calculated to obtain the actual content of metabolites in the samples.

Commercial standards for FA quantification are listed in Supplementary Table S3.

### 4.11. Data Analysis

All samples in the experiment were assessed in at least three independent biological replicates and all data are expressed as mean ± standard deviation (SD). Statistically significant differences between means were determined using the t-test or one-way analysis of variance (ANOVA) with a 5% significance difference. Graphs were constructed using Origin 9.5.1 (Microcal Software Inc., Northampton, MA, USA).

## 5. Conclusions

In conclusion, we have successfully obtained the *HrFAD2-1* and *HrFAD2-3* genes, which are not identical, and bioinformatic analyses revealed that they may belong to different classifications and perform different functions in seabuckthorn. Subcellular localization results were found to show that both *HrFAD2-1* and *HrFAD2-3* are localized in the endoplasmic reticulum. Using transient transformation of *Hippophae rhamnoides* L. fruits, we found that *HrFAD2-1* and *HrFAD2-3* may affect α-linolenic acid synthesis, with *HrFAD2-1* promoting the synthesis of lysophosphatidic acid. Both have a promoting effect on jasmonic acid (JA) synthesis and it is hypothesized that *HrFAD2* may improve plant stress tolerance. We obtained overexpression lines of *HrFAD2-1* and *HrFAD2-3* by the genetic transformation of *Linum usitatissimum* and found that *HrFAD2-1* and *HrFAD2-3* promoted plant growth, and that the *HrFAD2-1* gene was able to significantly increase the content of major fatty acids in *Linum usitatissimum* seeds.

## Data Availability

All analyzed data from this study are included in the content of this paper and in the Supplementary Materials.

## Supplementary Materials

The following supporting information can be downloaded at https://www.mdpi.com/article/10.3390/plants13223252/s1, Figure S1: Screening and phenotyping of transgenic plants; Table S1: All primer sequences related to this article; Table S2: Web sites for bioinformatics analysis; Table S3: FA Quantitative commercial standards.
