# Supplementary material for: The Molecular Cloning and Functional Analysis of the FAD2 Gene in Hippophe rhamnoids L
Source: Plants (Basel). 2024 Nov 20;13(22):3252. doi: 10.3390/plants13223252 (PMC11598821; doi:10.3390/plants13223252)
Supplement: Supplementary file 1 [file plants-13-03252-s001.zip › Supplementary Figure S1.pdf]

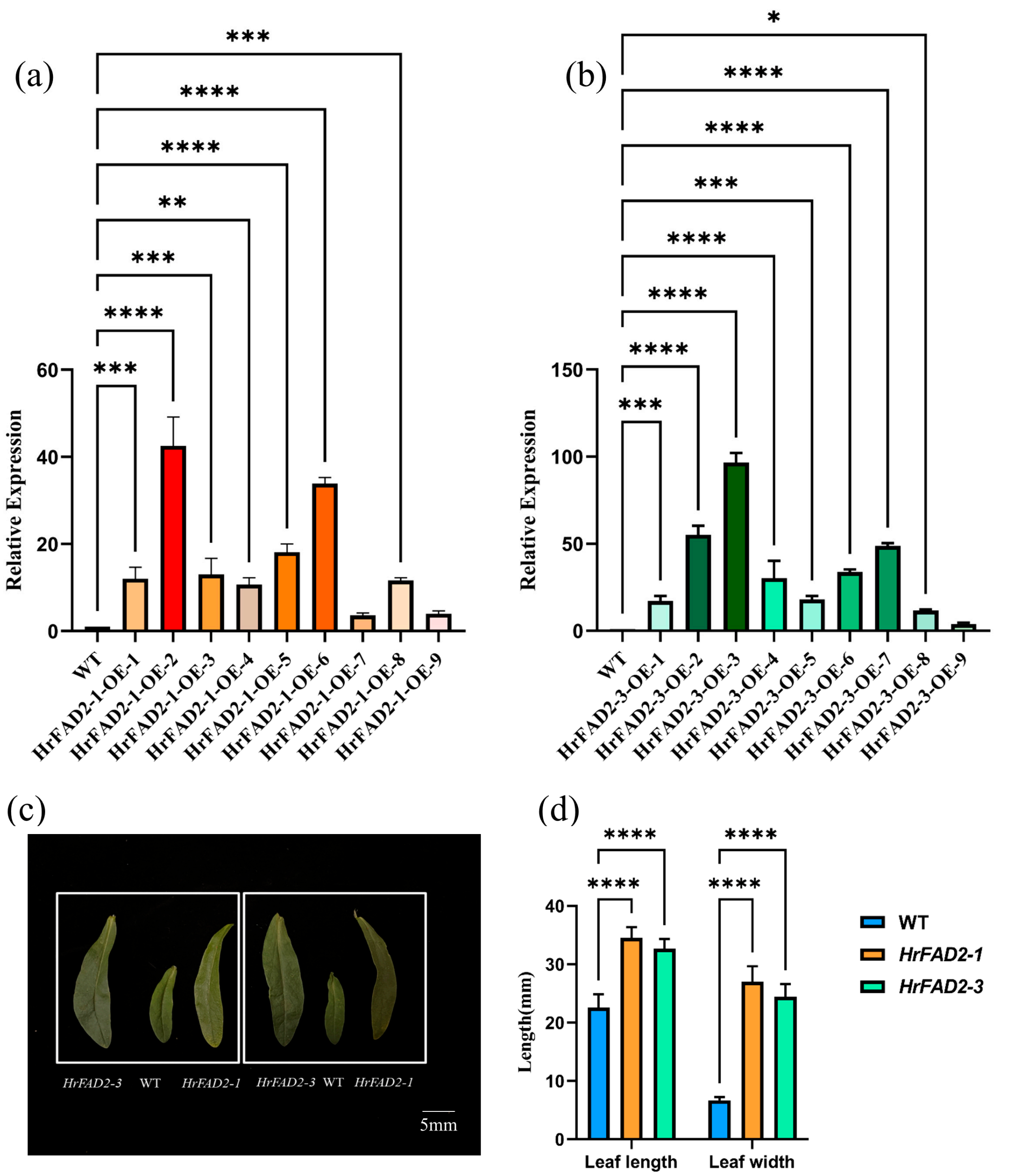

Supplementary Figure S1. Screening and phenotyping of transgenic plants. (a): Screening of *HrFAD2-1* transgenic plants. (b): Screening of *HrFAD2-3* transgenic plants. (c), (d): Observation of leaf phenotype of transgenic plants. (*：p＜0.05 **：0.001<P<0.01 ***：0.0001<P<0.001 ****: 0.00001<P<0.0001）
